# Primary mediastinal goiters

**DOI:** 10.1186/2193-1801-3-503

**Published:** 2014-09-07

**Authors:** Fayçal El Oueriachi, Mohamed Massine El Hammoumi, Adil Arsalane, Omar Slaoui, Hicham Diouri, El Hassane Kabiri

**Affiliations:** Department of thoracic surgery, Mohamed V Military Teaching Hospital, Appt 15, Imm 18, Jnane Nahda, Hay Nahda, Rabat, Morocco

**Keywords:** Ectopic goiter, Mediastinum, Surgery, Transthoracic approach

## Abstract

**Background:**

Primary mediastinal goiters (PMG) are very uncommon; few cases were reported in the literature.

**Patient description:**

We report here two cases of mediastinal goiters that met all criteria of PMG. Transternal approach was necessary for complete removal and pathological diagnoses confirmed their adenomatous goiter nature. The rarity of their occurrence, their clinical characteristics and surgical management were discussed.

**Conclusion:**

PMG is part of the differential diagnoses of mediastinal masses. Safe excision is ensured through transthoracic approach.

## Background

Ectopic goiters are very rare, they can be found from the tongue to the diaphragm. Ectopic location is related to the embryologic descent of the thyroid gland.

The majority of ectopic thyroid tissues (90%) are found in the base of the tongue. The rest (10%) may lie in the larynx, trachea, esophagus, mediastinum and heart.

PMG is a diagnostic challenge, which is frequently made after excision, often through transthoracic approach. Our objective is to report our modalities in the management of this very rare mediastinal disease through two cases of PMG from a total of 152 intrathoracic goiters operated in our department during 10 years from January 2004 to December 2013.

## Patient description

### Patient 1

**Figure 1:**
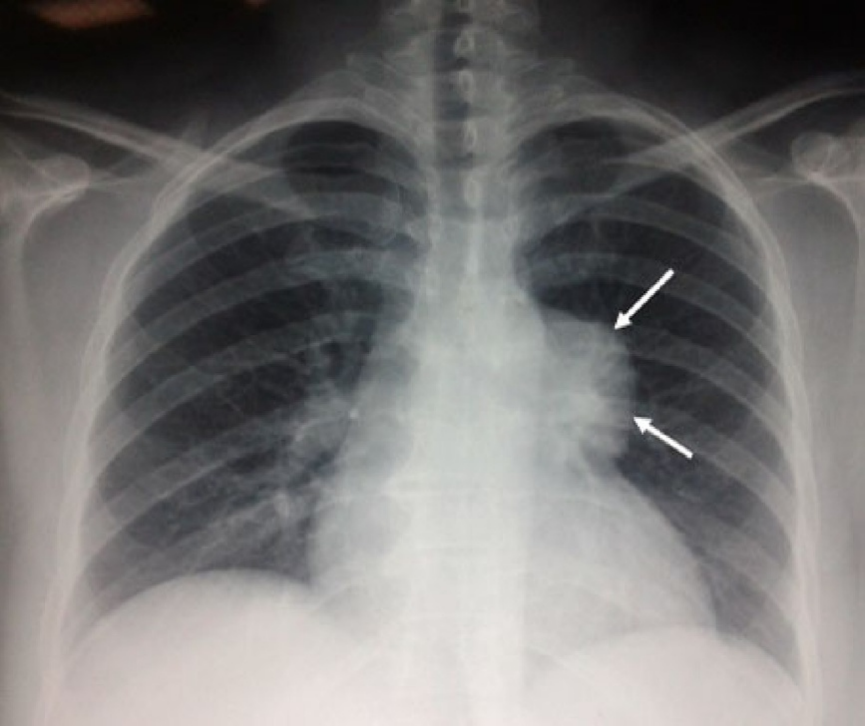
**Chest x-ray showing a large well defined opacity in the left hilar region.**

**Figure 2:**
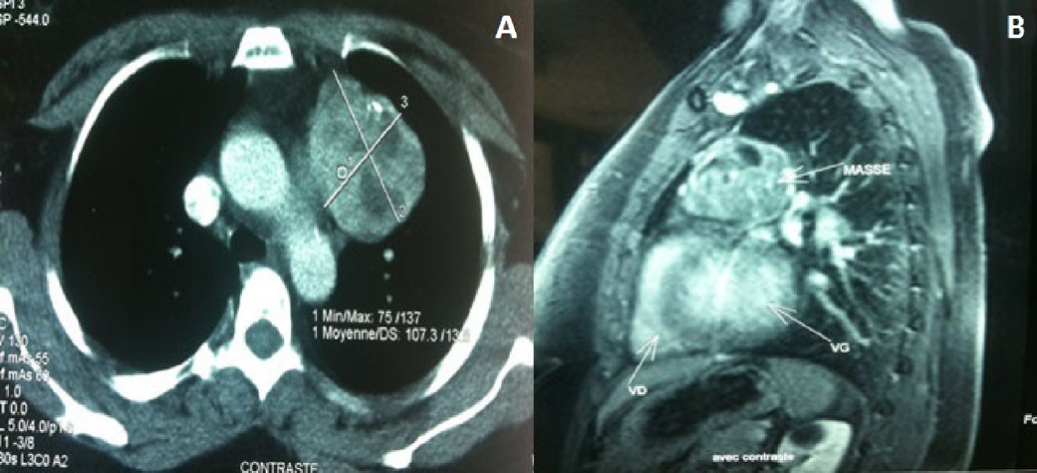
**Inhomogeneous multicystic mass lesion in the left mediastinum, tightly adherent to the aortic arch, pulmonary artery and left ventricle on CT scan (A) and MRI (B).**

A 50 year old woman presented with a five month history of palpitations. No cough, dyspnea or dysphagia were noted. She was clinically euthyroid. Physical examination showed an obese patient without cervical abnormalities. A chest X-ray (Figure 1) revealed a homogeneous opacity projecting on the left hilar region of the mediastinum. CT scan (Figure 2A) showed a 60 × 55 mm mass lesion with mixed density, containing microcalcifications and showing an enhancement after contrast iodine administration, in tight contact with the aortic arch, the pulmonary artery and the left ventricle. MRI (Figure 2B) demonstrated a multicystic mass (hypointense on T1 and hyperintense on T2), with hemorrhagic areas (hyperintense T1 and T2) and calcifications (hypointense T1 and T2). Referring to the CT scan and MRI findings, the description was compatible with a suspected preoperative diagnosis of teratoma or primary intrathoracic goiter. Other mediastinal tumors such as thymoma could not be ruled out. Laboratory tests showed normal thyroid function and normal βHCG and αFP levels. Thyroid ultrasonography was normal.

**Figure 3:**
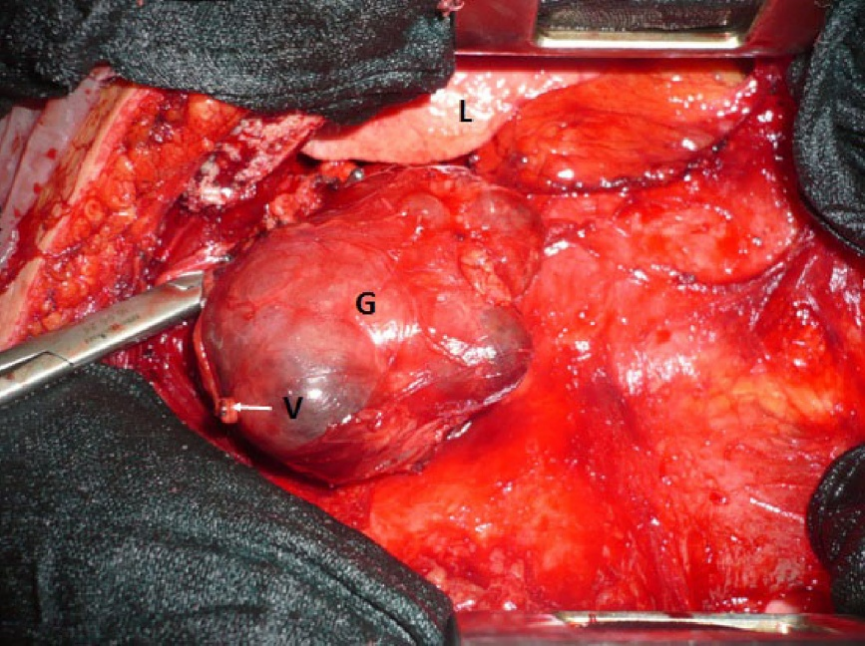
**Operative view; L: lung; G: goiter; V: ligated mediastinal vessel.**

The tumor was completely resected through a total median sternotomy (Figure 3). There were no tissular or vascular connections between the mass and the cervical thyroid gland, the feeding artery was dependent on the brachiocephalic artery and other intrathoracic small vessels. The histopathological findings showed a multinodular colloid goiter without signs of malignancy. The postoperative course has been marked by the appearance of dyspnea related to pleural and pericardial effusions treated by pleural drainage and pericardial puncture. Forty two months after surgery, the patient was asymptomatic and disease free.

### Patient 2

**Figure 4:**
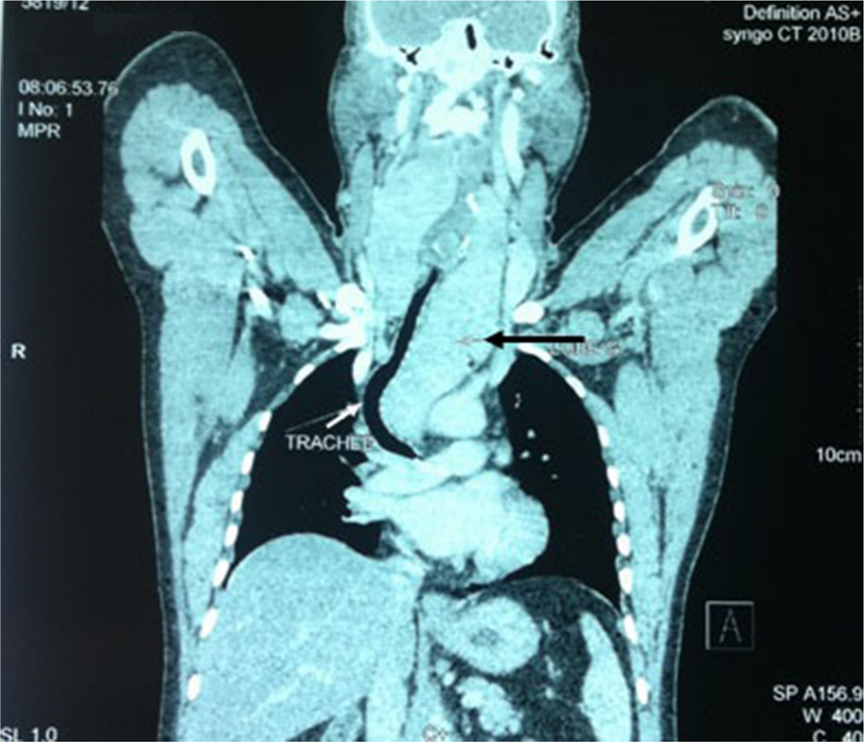
**Coronal CT scan showing a giant goiter extending into the mediastinum (black arrow), laminating and deflecting the trachea to the right (white arrow).**

**Figure 5:**
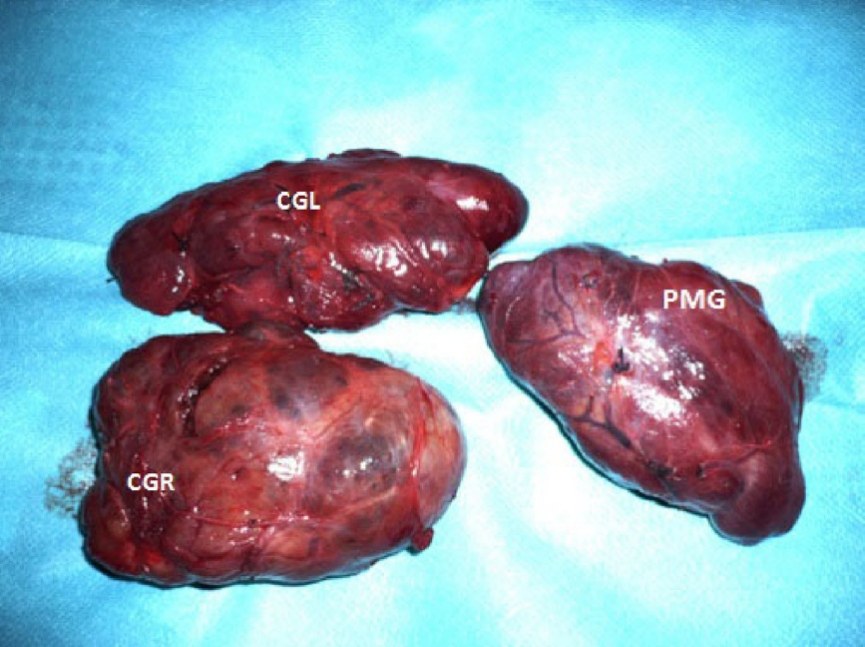
**The examination of the two specimens reveals that they have the same macroscopic appearance.** No connections between the two masses were found. CGL: left lobe of the cervical goiter; CGR: right lobe of the cervical goiter; PMG: primary mediastinal goiter.

A 55 year old man was referred to our department for surgical management of a substernal goiter. He had a 7 year history of a slowly growing anterior neck mass, associated during the last year with a dry cough, shortness of breath, dyspnea, dysphonia, palpitations and sweating. He was followed by an endocrinologist for hyperthyroidism, for which he was placed on neomercazole for thyroid suppression. Physical examination showed an enlargement of the thyroid gland; it was bilateral, irregular and non adherent. The inferior pole of the goiters left lobe was not palpated because of its extension through the thoracic inlet. Chest X-ray showed that upper mediastinum was enlarged and the trachea right deviated. An ultrasound examination of the neck had revealed a multinodular goiter which the left lobe extends inside the thorax. CT scan of the chest (Figure 4) demonstrated a large multinodular goiter which left lobe measuring 20 × 5,2 × 4,4 cm, it enlisted in the anterior mediastinum laminating the trachea and displacing it to the right. Its lower pole down into the middle mediastinum and arrives below the carina. The diagnosis of substernal goiter was made, and the patient consented for total thyroidectomy through Transcervical approach, and if necessary transthoracic approach. At first, a thyroidectomy was performed via standard neck approach. The presumed intrathoracic part of the goiter was easily extracted after ligation-section of the inferior left pedicle. After excision of the goiter we found that the left lobe was well encapsulated and measured 12 cm in length, this remark was inconsistent with CT findings. The digital exploration followed by axial mediastinoscopy of the anterior mediastinum noted the presence of a residual mediastinal mass. Total median sternotomy was then performed. Intraoperative findings revealed that the mass was totally separated from the cervical goiter and its blood supply was independent from thyroid vessels. This mediastinal mass had macroscopic similarities with the cervical goiter (Figure 5). It derived its blood entirely from thoracic vessels, especially from the ascending aorta. The patient had an uneventful postoperative recovery and was discharged from hospital after 6 days. He was put on daily thyroid substitution. The histopathological examination showed the same diagnosis of multinodular colloid goiter with no malignancy in the two specimens. Eighteen months after surgery, the patient was asymptomatic and disease free.

## Discussion

Primary mediastinal goiters are considered in Rives classification established in 1947 (Rives 1947) as an aberrant goiter. The precise description of PMG is defined as the presence in the mediastinum of thyroid tissue that met the following criteria: the cervical thyroid gland can be present or absent with no history of thyroidectomy, there is a total discontinuity between the cervical thyroid and the mediastinal goiter, the mediastinal mass receives its blood supply from the mediastinal vessels and no history or evidence of malignancy is documented in both cervical and mediastinal goiters (Shields 1995; Foroulis et al. 2009). Only up to 1% of all substernal goiters fulfill the above criteria, it’s why PMG is an extremely rare entity. In our experience, from 152 substernal goiters managed in our department, only two cases were a PMG; our findings are consistent with the literature. As far as we know, less than ten cases of PMG were reported (Thuilier & Venot 2012).

In our first case, the patient had a PMG. In the second case, the separation of the mediastinal goiter from the substernal part of the cervical thyroid and its blood supply exclusively from mediastinal vessels were clearly visible intraoperatively; the patient had an exceptional association between a primary and secondary MG, it’s the first case described in the literature.

The primordial thyroid gland is first identifiable during the fourth week of gestation on the tongue; it begins its descent during the fifth week to reach its final position, anterior to the trachea, in the eighth week. Ectopic thyroid tissue may occur at any point along this pathway. In rare conditions, the totality or part of thyroid tissue may descend into the thorax drawn by the caudal migration of the heart. Few cases of ectopic thyroid were reported to lie in the mediastinum, the trachea, the esophagus, the thymus and the heart (Spinner et al. 1994; Arriaga & Myers 1988).

Symptoms related to PMG are often those indicating a compression of adjacent structures, such as dysphagia, dyspnea, hoarseness or superior vena cava syndrome. Thyrotoxicosis was rarely described. The majority of patients with PMG are asymptomatic; their tumors were reported as incidental findings on chest roentgenogram or chest CT scan (Shah et al. 2007a). In our patients, symptoms were present, related to the compression of adjacent structures due to the large size of the tumor in the two cases.

Plain chest roentgenogram shows the shadow of mediastinal mass in 70%, presence of calcifications is highly suggestive. The sensivity of ultrasound in the evaluation of PMG is poor. However, in substernal position CT scan and MRI are very effective. Chest CT scan provides information pointing toward the thyroid nature of the mediastinal mass (Senac & Giron 1992). Heterogeneous multicystic appearance, presence of calcifications, intense enhancement following intravenous administration of iodine and the discontinuity between the mediastinal mass and the cervical thyroid gland are characteristic signs of PMG. Findings on MRI are comparable to the CT (Pappalardo et al. 1993). Calcifications are not clearly visible on MRI, but the relations with great vessels and adjacent organs are best assessed.

Although rare, ectopic thyroid tissue might be malignant. Therefore iodine containing contrast medium might delay further possibility of radioiodine treatment, I123 scan or MRI might be a reasonable alternative to CT scan. This should be taken into consideration, when thoracic mass is detected by X-ray.

Thyroid scintigraphy is not accurately used in the diagnosis of mediastinal masses. When performed, it’s effective for differential diagnosis of other mediastinal tumors including lymphadenopathy, thymoma, teratoma, bronchogenic cysts and primary tracheal or esophageal tumors.

In our two patients CT was sufficient to suggest the diagnosis of MG. MRI was performed in the first case to better plan the surgical technique.

Surgical resection is the gold standard in the treatment of PMG. Its role is both diagnostic and therapeutic; it allows ensuring the diagnostic by providing tissue for histological study, it also allows to rule out malignancy and to remove compression of adjacent structures, such as trachea and heart to prevent the risk of tracheomalacia and heart rhythm disorders. The risk of postoperative tracheomalacia and consequent tracheostomy is slightly increased but occurs very rarely, it’s especially seen in long standing and large goiters with tracheal compression. In our second patient, the trachea was compressed severely, but after the removal of the two masses, it regained its normal aspect and position without chondromalacia.

Resection is often achieved through median sternotomy or thoracotomy to ensure all vessels supplying the mass are adequately ligated. Thoracotomy is potentially required when the mediastinal mass is lateralized to the left or the right side. In the first case, the goiter was lateralized to the left, but we preferred sternotomy because the mass had an intimate contact with the heart and great vessels. The combination of cervical incision and sternotomy is useful in excision of mediastinal masses associated with cervical goiter (Tang et al. 2002). A wide surgical access through complete median sternotomy was our choice in the two cases because of the large size of the goiters and their deep location into the mediastinum. The extraction of PMG through cervical approach should be prohibited because of the risk of intrathoracic hemorrhage and the difficulties to control it through this approach (Hall et al. 1988; Falor et al. 1963).

Thoracoscopy has been described as a new technique in resection of mediastinal masses (Grondin et al. 2001). In selected patients; improved visibility and low morbidity make thoracoscopy a technique of choice.

In conclusion, we reported two cases of PMG that highlight the need to keep in mind that ectopic goiter, although rare, is part of the differential diagnoses of mediastinal masses. Transthoracic approach is mandatory for safe excision. The precise histological diagnosis of PMG is often obtained after surgical excision of the tumor.

## Abbreviations

PMG: Primary mediastinal goiter
αFP: Alpha fetoprotein
βHCG: Beta chorionic gonadotropin hormone.
